# Decreased monocyte shedding of the migration inhibitor soluble CD18 in alcoholic hepatitis

**DOI:** 10.1038/s41424-018-0022-7

**Published:** 2018-06-15

**Authors:** Sidsel Støy, Thomas Damgaard Sandahl, Anne Louise Hansen, Bent Deleuran, Thomas Vorup-Jensen, Hendrik Vilstrup, Tue Wenzel Kragstrup

**Affiliations:** 1 Department of Hepatology and Gastroenterology, Aarhus University Hospital, Aarhus, Denmark; 2 Department of Biomedicine, Aarhus University, Aarhus, Denmark; 3 Department of Rheumatology, Aarhus University Hospital, Aarhus, Denmark

## Abstract

**Objectives:**

During alcoholic hepatitis (AH) monocytes traverse the vascular boundaries and massively invade the liver. In principle, tissue extravasation can be limited through shedding of CD18 integrins from leukocytes, including monocytes. The soluble (s) product sCD18 conceals adhesion receptors on the endothelium, which reduces monocyte extravasation. In AH, monocytes are dysfunctional, but whether this involves their self-generated anti-migration is unknown. Our aim was, therefore, to investigate monocyte CD18 dynamics in AH.

**Methods:**

We studied 50 AH patients and 20 healthy controls. We measured monocyte expression and conformational activation of CD18, plasma (P)-sCD18, stimulated in vitro CD18 shedding and P-sCD18 in a short-term chronic-binge mouse model.

**Results:**

AH-derived monocytes had a 30–60% higher expression of active CD18 receptors (*p* < 0.01), but the sCD18 concentration per monocyte was reduced in vivo by 30% and in vitro by 120% (*p* < 0.01). Ethanol reduced the in vitro shedding of CD18 in the patients only. TNFα increased sCD18 concentration per monocyte, but less so in the patients (*p* < 0.04). P-sCD18 per monocyte was inversely related to disease severity. In early alcoholic liver disease, P-sCD18 was decreased in the mouse model.

**Conclusions:**

The monocyte CD18 integrins are highly activated in AH and the single monocyte shedding of CD18 was decreased favoring tissue extravasation. Alcohol in itself and altered monocyte responsiveness to TNFα may explain this lowered shedding.

**Translational impact:**

The contribution of this mechanism to the excessive monocyte liver infiltration in AH should be further explored as it may serve as a potential therapeutic target to limit liver inflammation.

## Introduction

Alcoholic hepatitis is the most severe type of alcoholic liver disease associated with high morbidity and mortality. The lack of effective treatment options in these patients underlines the need for a better pathophysiological understanding of the disease.

In alcoholic hepatitis, neutrophil liver infiltration is a hallmark, but also monocyte and macrophage numbers in the liver increase dramatically and contribute towards the florid inflammatory state^1,2^. Circulating monocytes may be divided into subsets based on their expression pattern of CD14 and CD16, with the CD14^+^CD16^+^, intermediate monocytes, having an inflammatory phenotype and being the subset most predominantly recruited during inflammation^3–5^. The CD18 (β2) family of heterodimeric integrins, comprising CD11a/CD18, CD11b/CD18, CD11c/CD18 and CD11d/CD18, are central for leukocyte extravasation from blood into tissues. CD18 integrins require conformational changes to bring the receptors to an active state with high affinity for ligands^6^. This can be detected on the cell surface by conformation-sensitive antibodies^7^.

CD11a/CD18 and CD11b/CD18 are expressed on monocytes and mediate adhesion to the endothelium via intercellular adhesion molecule (ICAM)-1 as part of the extravasation process. In alcoholic hepatitis, ICAM-1 is up-regulated in the liver along with the presence of a CD18 positive inflammatory infiltrate^8^. In ICAM-1 knock-out mice, leukocyte recruitment to the liver is reduced in models of alcoholic liver injury and also antibodies targeting CD18 reduce liver injury in these models^9–12^. This supports a dominant role of this system in alcohol induced liver inflammation.

Recently, soluble (s) complexes of the CD18 integrin receptor have been discovered and their plasma concentration shown to change as part of inflammatory diseases^13^. TNFα can induce shedding of CD18 in vitro leading to the production of sCD18 complexes^14–16^. In the circulation of alcoholic hepatitis patients, levels of pro-inflammatory cytokines such as TNFα are elevated^17^. The sCD18 complexes antagonize monocyte adhesion to ICAM-1 in a concentration-dependent manner and also promote macrophage efflux from sites of inflammation^14,18^. Shedding of the soluble complex may thus be a tissue inflammation-limiting mechanism. However, monocytes from patients with alcoholic hepatitis are dysfunctional in their production of reactive oxygen species and their killing of bacteria^19^. We hypothesized an up-regulation of active CD18 receptors on monocytes in patients with alcoholic hepatitis and that their shedding of CD18 is defective, which together could potentially promote and aggravate hepatic inflammation in AH.

Following this hypothesis, we investigated the expression of activation-dependent epitopes in monocyte CD18 in patients with alcoholic hepatitis and measured the levels of sCD18. The findings were related to clinical characteristics. To support a mechanistic understanding, we compared the influence of ethanol, TNFα and lipopolysaccaride. Our findings now highlight previously unanticipated aspects of CD18 integrin activation and shedding in inflammatory disease, namely the dysregulation of these molecular mechanisms in alcoholic hepatitis.

## Materials and methods

### Study design and population

In this prospective cohort study, we consecutively recruited 50 patients with alcoholic hepatitis from four hospitals in central Denmark. We included 20 age-matched healthy persons with no previous history of liver disease as controls. The patients were followed for 30 days with samples obtained at the day of diagnosis and day 14 and day 30 after diagnosis. Between diagnosis and day 14 four patients died and five were lost to follow-up or denied ongoing participation. Between days 14 and 30, the numbers were two and eight, respectively. Patients were stratified using the Glasgow Alcoholic Hepatitis Score (GAHS). Patients with GAHS ≥ 9 on admission were treated with 400 mg pentoxifyllin t.i.d. in accordance with Danish national guidelines at time of recruitment in addition to nutritional therapy. Patients with GAHS < 9 were treated solely with nutritional support and standard medical care. The pentoxifyllin-treated patients do not differ significantly from the un-treated patients with regards to clinical and biochemical characteristics. No patient received prednisolone. Patient characteristics are presented in Table 1 for all alcoholic hepatitis patients.

**Table 1:** Patient characteristics

	Alcoholic hepatitis		
	Days after diagnosis		
	Day 0	Day 14	Day 30
Gender: F/M	15/35	13/28	11/20
Age (years)	53±8	53±7	52±7
Weight (kg)	76±16	76±16	75±16
BMI	25±4	25±4	25±4
Alanine aminotransferace (10–45 U/l)	66±77	92±70	76±60
Bilirubin (5–25 μmol/l)	299±168	179^**^±141	107^**^±140
Alkaline phosphatase (10–75 U/l)	252±149	231±159	200±134
Sodium (137–145 mmol/l)	131±7	134^**^±5	134±5
Creatinin (45–90 μmol/l)	75±40	76±56	62±18
Albumin (36–45 g/l)	27±5	30^*^±6	33^*^±6
Hemoglobin (7.3–9.5 mmol/l)	6.7±0.96	6.9±1.1	7.0±1.2
Thrombocytes (165–400 × 10^9^/l)	142±67	152±108	188±100
International normalized ratio (<1.2)	2.0±0.6	1.7^*^±0.6	1.5^*^±0.5
C-reactive protein (<75 mmol/l)	337±192	233^*^±196	168±181
Leukocytes (3.50–10.0 × 10^9^/l)	12.8±9.00	14.7^*^8.8	13.6±9.9
Monocytes (0.20–0.70 × 10^9^/l)	1.4±0.7	1.0^*^±0.6	1.0±0.6
MELD	21±7.2	16^**^±9	12^**^±7
Child Pugh Score	10.9±1	9.7^**^±1.8	8^**^±2
Glasgow Alcoholic Hepatitis Score	8.8±2	8^*^±2	7^**^±2
MDF	72±35	49^**^±41	33^*^±30

Clinical and biochemical characteristics of the alcoholic hepatitis patients are presented at the days 0, 14 and 30 after diagnosis in accordance with the sampling days. Data are expressed as mean ± SD. The study days are compared with a paired *T*-test (day 0 with day 14 and day 14 with day 30

*p  <  0.05, **p >  0.01

*MELD* model of endstage liver disease, *MDF* modified Maddrey discriminant function

### Inclusion and exclusion criteria

Patients between the age of 18 and 75 years were included when diagnosed with alcoholic hepatitis in similar manner to the criteria later formulated in the established guidelines^20^. The diagnosis was based on a set of clinical and biochemical variables; a history of excessive alcohol consumption with less than 3 weeks of abstinence leading up to admission; acute jaundice (developed within the previous 2 weeks with serum bilirubin >80 μmol). Patients were excluded if they had biliary stones, other liver diseases, gastrointestinal bleeding within the past 3 months, signs of an infectious focus (clinical signs or evidence on abdominal ultrasound, chest X-ray, urine samples or ascites), hepatocellular carcinoma or other malignancy or had received immune-modulating therapy within the 8 weeks leading up to admission. When in diagnostical doubt biopsy was performed (*n* = 10) and none of these refuted the diagnosis.

### Consent and data collection

The study was conducted in accordance with the Helsinki Declaration and approved by the Central Denmark Region ethical committee (j.no. 20100281). Informed, written consent was obtained prior to study inclusion. The study was registered at ClinicalTrials.gov (NCT00992888). Clinical and biochemical data were collected on all study days. Disease severity was scored by GAHS, Modified Maddrey Discriminant function, Model of Endstage Liver disease (MELD) and Child Pugh Score.

### Blood sampling

Venous sampling was performed in EDTA vacuum tubes. Peripheral blood mononuclear cells (PBMCs) were isolated within 2 h of sampling using Ficoll–Hyperpaque centrifugation (GE Healthcare Bio-sciences, Uppsala, Sweden) and stored at −140 °C. Plasma was obtained from centrifugation of whole blood 3000 g, 10 min at 4 °C and stored at –80 °C for later side-by-side analyses.

### Flow cytometric analyses of PBMCs

PBMCs (1 × 10^6^ cells/setup) were blocked for unspecific binding in phosphate-buffered saline with 1 % (w/v) bovine serum albumin (Calbiochem, San Diego, CA, USA) together with 10 μg/ml murine IgG (Jackson ImmunoResearch, PA, USA). PBMCs were stained with either 10 µg/ml biotinylated IgG1 antibody to CD18 (KIM127) or 10 µg/ml biotinylated IgG1 isotype control in combination with secondary antibody streptavidin-FITC (Dako). The KIM-127 antibody to CD18 is an established marker for the ligand-binding active conformation of CD18 integrins^7^. The following antibodies were used for leukocyte subset stratification of the PBMCs: anti-CD18 BV421 (BD Horizon), anti-CD14 V500 (BD Horizon), anti-CD16 PE/Cy7 (Biolegend). Anti-CD56 APC-eFlour780 (eBioscience) and LiveDead staining (Near-IR; Invitrogen) were used for CD56^−^dump (exclusion of natural killer cells) and viability estimation. All samples were analyzed immediately after staining using BD LSRFortessa Cell Analyzer. Data analyses were performed in FlowJo (Tree Star Inc., Ashland, OR, USA). Monocytes were divided into three subsets based on their expression of CD14 and CD16; classical (CD14^+^CD16^−^), intermediate (CD14^+^CD16^+^) and non-classical (CD14^lo^ CD16^+^) (supplementary figure 1). This assay was performed on samples from 15 patients and 8 controls.

### Plasma measurements of sCD18 and sCD163

In house TRIFMA assays where utilized to measure sCD18 in human plasma and mouse plasma as previously published^21^. In- house ELISA assays were utilized to measure sCD163 in human plasma as previously described^22^. Plasma lipopolysaccharide (LPS) levels were measured using the Limulus amebocyte lysate assay (HIT302, Hycult Biotec, Uden, the Netherlands). When correcting for the number of monocytes, total leukocytes and neutrophils, we appointed the healthy controls a value of 0.45 × 10^9^, 6.75 × 10^9^ and 4.5 × 10^9^/l, respectively, i.e. the mean of the reference intervals. In patients with alcoholic hepatitis we used leukocyte differential counts collected on all study days.

### In vitro culture experiments with PBMCs

For *in vitro* culture experiments with PBMCs, cells (4 × 10^5^ cells/setup) were cultured in RPMI medium supplemented with 10% (v/v) heat-inactivated FCS (Sigma-Aldrich, St. Louis, MO, USA), 1 % (v/v) penicillin–streptomycin–glutamine (Life Technologies, CA, USA). PBMCs were stimulated with either 10 ng/ml TNFα (PeproTech, NJ, USA), 10 ng/ml LPS (Sigma-Aldrich, MO, USA), 25 mmol/l ethanol (Sigma-Aldrich) or left untreated for 48 h at 37 °C in a humidified incubator with 5% CO_2_. After incubation, supernatants were stored at −80 °C.

### Chronic-binge mouse model

We employed the National Institute on Alcohol Abuse and Alcoholism (NIAAA) chronic-binge model of ethanol mediated injury^23^. Histological haematoxylin-eosin and masson trichrome stains were performed on paraffin embedded tissue sections and markers of liver injury (ALT, AST) were measured in plasma to verify the development of liver injury. Plasma was stored for later sCD18 analyses. The study was approved by the national animal ethics committee (protocol no. 2013-15-2934-00872/BES) and conducted in accordance with local and international animal welfare guidelines.

### Statistics

We utilized non-parametric testing of our experimental data. Analyses of differences between groups, alcoholic hepatitis and healthy controls, were carried out by Wilcoxon rank-sum test and Wilcoxon signed-rank test was used for paired data. Patient characteristics, which were normally distributed, were compared using parametric tests. We used Spearmans rho for correlation analyses. Results are presented as median (interquartile range) unless specified otherwise, and a two-tailed *p*-value < 0.05 was considered statistically significant.

## Results

### Monocytes have higher expression of active CD18 in alcoholic hepatitis

**Fig. 1:**
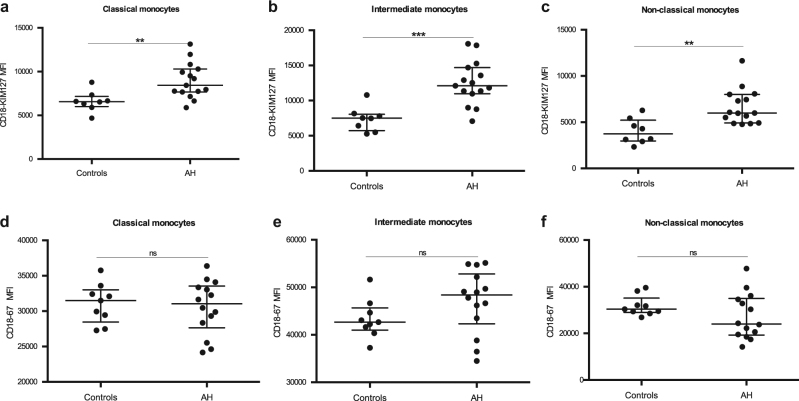
Increased expression of activated CD18 on monocytes in alcoholic hepatitis. By flow cytometry, we measured CD18 expression using two different antibodies detecting the ligand-binding active (**a**–**c**) (CD18 KIM127) and (**d**–**f**) total CD18 (CD18-67) on monocyte subpopulations from peripheral blood mononuclear cells. Monocyte subpopulations were identified as follows; classical (CD14^+^CD16^-^), intermediate (CD14^+^CD16^+^), non-classical (CD14^lo^CD16^++^). The monocyte expression of CD18 is expressed as median fluorescence intensity (MFI). Expression in alcoholic hepatitis (AH) patients is compared with the expression in healthy controls (Controls). Ranksum, bars represent median, interquartile range, **p < 0.01, ***p < 0.001, ns not significant

In all three monocyte subsets examined, we observed an increased expression of the ligand-binding active conformation of CD18 in alcoholic hepatitis in comparison with the expression in healthy controls (Fig. 1) We also measured the expression of total CD18 to investigate whether our findings reflected an increased activation or a general up-regulation of the receptor complex. We found no difference in the expression levels of total CD18 between the two groups in any of the monocyte subsets.

### The shedding of CD18 per monocyte is reduced in alcoholic hepatitis

**Fig. 2:**
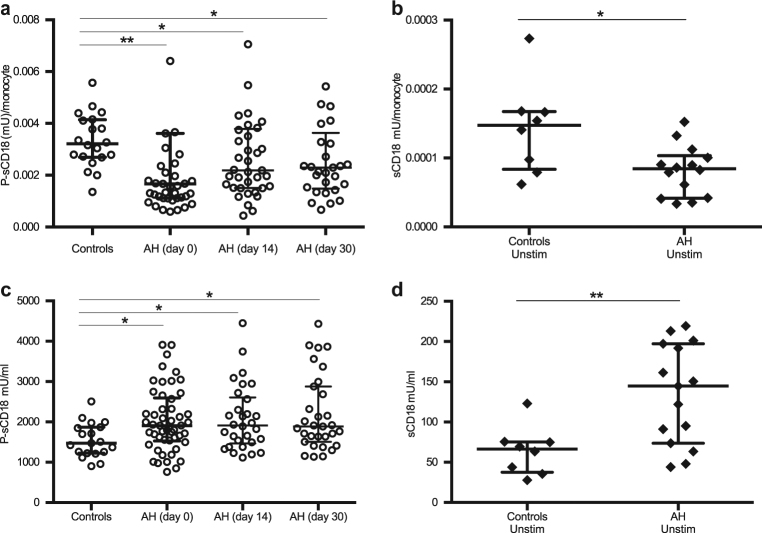
Decreased monocyte shedding of CD18 in alcoholic hepatitis. **a** The plasma (P) concentration of sCD18 was measured by TRIFMA in healthy controls and in patients with alcoholic hepatitis at the day of diagnosis (day 0) and at day 14 and 30 after diagnosis and divided by monocyte count. **b** Peripheral blood mononuclear cells were cultured 48 h at 37 °C without stimulation and the concentration of sCD18 was measured in the supernatant and divided by monocyte count. **c** Total P-sCD18 and **d** total in vitro sCD18. Alcoholic hepatitis (AH) patients are compared with healthy controls (Controls), ranksum, bars represent median, interquartile range, *p < 0.05, **p < 0.01

We assessed whether the elevated surface expression of CD18 was accompanied by a higher plasma (P)-sCD18 in alcoholic hepatitis. Because the patients with alcoholic hepatitis had elevated numbers of monocytes in their peripheral blood (normal ref. interval 0.2 to 0.7 10 × 10^9^/l) (Table 1), we compared the sCD18 concentration per monocyte between the groups. P-sCD18 per monocyte was significantly lower in alcoholic hepatitis at the day of diagnosis (Fig. 2a, *p* < 0.01). The level stayed low throughout the 30 days’ follow-up. Other leukocyte subsets especially neutrophils may also contribute to the plasma pool of sCD18. As both the neutrophil count and the total leukocyte count was elevated in the alcoholic hepatitis patients, we assessed whether taking this into account changed the pattern. Indeed, both P-sCD18 per neutrophil and P-sCD18 per leukocyte were reduced in alcoholic hepatitis compared with healthy controls (*p* < 0.05) (supplementary figure 2). To investigate whether the decreased P-sCD18 per monocyte could be explained by lowered shedding, we cultured PBMCs and measured their spontaneous shedding of CD18. Consistently, the spontaneous CD18 shedding per monocyte was lower in alcoholic hepatitis (Fig. 2b, *p* < 0.05). The corrected P-sCD18 per monocyte values did not correlate with monocyte CD18 expression.

### Elevated levels of total P-sCD18 and total in vitro sCD18 concentration in alcoholic hepatitis

The total concentration of P-sCD18 was 30% higher in patients with alcoholic hepatitis than in healthy controls (Fig. 2c, *p* < 0.05). The concentration of sCD18 did not change significantly during the 30 days follow-up period and continued to be higher than levels in healthy controls (*p* < 0.05). PBMCs from patients with alcoholic hepatitis likewise spontaneously produced more sCD18 than cultures of healthy PBMCs (Fig. 2d, *p* < 0.01). There were no correlations between the monocyte surface expression of CD18 and P-sCD18 or CD18 shedding in vitro. However, the frequency of circulating intermediate monocytes tended to correlate with P-sCD18 (*r* = 0.48, *p* = 0.07) and with spontaneous in vitro sCD18 concentration (*r* = 0.45, *p* = 0.09).

### Macrophage activation is related to sCD18 formation in alcoholic hepatitis

We observed a positive correlation between the plasma marker of macrophage activation; sCD163, and sCD18 in patients with alcoholic hepatitis (*r* = 0.49, *p* = 0.0002) (data not shown). The correlation was present also at day 14 and day 30 after diagnosis.

### Ethanol decreases CD18 shedding only in alcoholic hepatitis patients

**Fig. 3:**
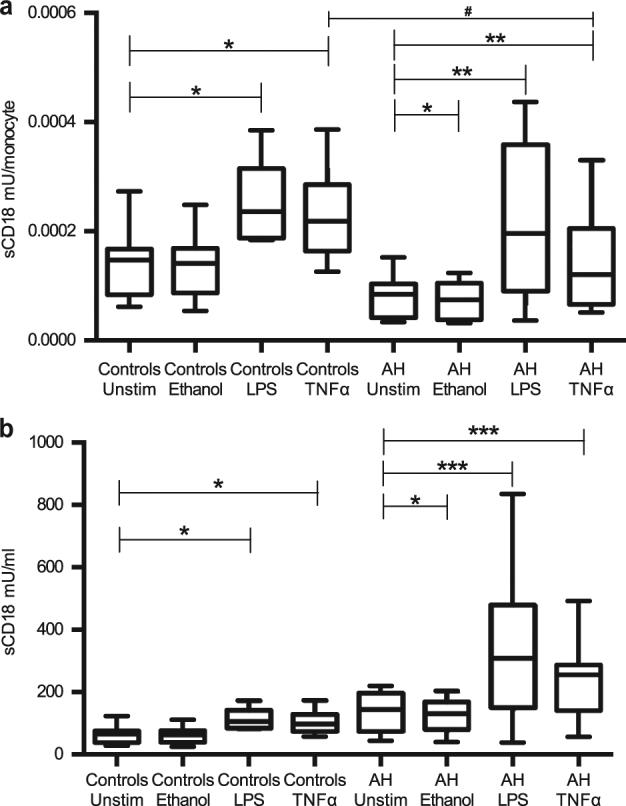
In vitro shedding of CD18 by ethanol, TNFα and LPS in alcoholic hepatitis. Peripheral blood mononuclear cells from alcoholic hepatitis patients and healthy controls were cultured without stimulation or with ethanol 25 mmol/l, TNFα 10 ng/ml or lipopolysacaride (LPS) 10 ng/ml for 48 h at 37 °C. Supernatants were collected and sCD18 concentration was measured by TRIFMA. **a** The stimulation induced sCD18 concentration was divided by monocyte count and compared with spontaneous shedding within each groups. The TNFα-induced shedding was compared between the groups. **b** Total stimulation induced sCD18 concentration was compared to unstimulated within each group. Signrank, boxes represent median, interquartile range. *p < 0.05, **p ≤ 0.01, ***p < 0.001. Ranksum, ^#^*p* < 0.05

We examined which mediators could be responsible for moderating the shedding of CD18 in alcoholic hepatitis. Interestingly, ethanol only had an impact on CD18 shedding in alcoholic hepatitis patients. In these patients, it reduced CD18 shedding per single monocyte as well as the total sCD18 level (Fig. 3a, b, *p* < 0.05). TNFα and LPS was able to increase the shedding of CD18 per monocyte and the total sCD18 level both in alcoholic hepatitis and in healthy controls (Fig. 3a, b, *p* < 0.05). However, the TNFα-mediated enhancement of CD18 shedding per monocytes was lower in alcoholic hepatitis patients than in healthy controls (*p* < 0.05). LPS increased CD18 shedding per monocyte equally in alcoholic hepatitis patients and in healthy controls. P-LPS levels correlated with P-sCD18 levels in patients with alcoholic hepatitis (*r* = 0.30, *p* = 0.03).

### Shedding of CD18 correlated with clinical scores

We looked at correlations between sCD18 and clinical measures at diagnosis. The correlations between P-sCD18 per monocyte and disease scores were inverse (GAHS *r* = −0.19, *p* = 0.089, MELD *r* = −0.24, *p* = 0.12). P-sCD18 correlated with Child Pugh Score (*r* = 0.41, *p* = 0.003) and tended to correlate with GAHS (*r* = 0.24, *p* = 0.1) and MELD (*r* = 0.20, *p* = 0.18). P-sCD18 also correlated with ascites (*r* = 0.31, *p* = 0.02) and pp (*r* = −0.31, *p* = 0.03). The spontaneous shedding of CD18 correlated positively with GAHS (*r* = 0.56, *p* = 0.04) and tended to also correlate with Child Pugh Score (*r* = 0.41, *p* = 0.1). Monocyte expression of CD18 was not related to disease scores at diagnosis. When comparing the change in P-sCD18 per monocyte with the change in disease scores through the 30 days follow-up, we observed an increase in P-sCD18 per monocyte with decreasing GAHS (*r* = −0.50, *p* = 0.01) and a similar tendency for Child Pugh Score (*r* = −0.33, *p* = 0.10) and MELD (*r* = −0.26, *p* = 0.20). P-sCD18 did not correlate with changes in disease scores.

### Similar findings in pentoxifyllin-treated and un-treated patients

As TNFα induce the shedding of CD18, pentoxifyllin treatment may theoretically lower P-sCD18 by reducing shedding, and this could affect our results. We therefore tested whether P-sCD18 at diagnosis was different comparing pentoxifyllin treated to un-treated patients and found no difference (1930 ± 1460 vs. 1907 ± 652, *p* = 0.65). We also verified that performing our analyses on the pentoxifyllin treated and the un-treated patients separately did not change our results on P-sCD18, monocyte expression of CD18 or our in vitro findings.

### Plasma levels of sCD18 are lowered in a mouse model of ethanol-induced liver injury

**Fig. 4:**
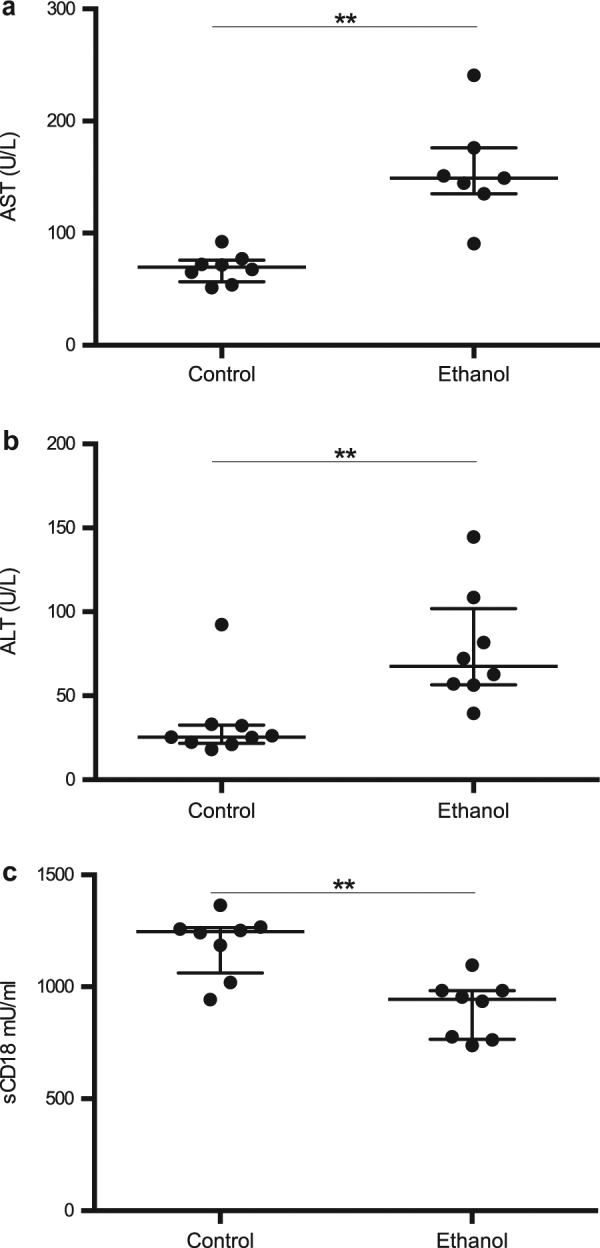
Plasma sCD18 is deceased in a short-term model of alcohol induced liver injury. Using female C57/BL mice, we employed the chronic-binge ethanol model of alcoholic liver injury. Mice were fed a liquid Lieber-DeCarli diet containing either ethanol or maltose dextrin (control for calories in ethanol) for 10 days and gavaged with ethanol or maltose dextrin at day 10 and sacrificed 9 h later. **a** Asparate transaminase (AST) and **b** alanine transaminase (ALT) were measured in plasma. (**c**) Plasma levels of sCD18 were measured by TRIFMA and the levels compared between the groups. Ranksum, bars represent median and interquartile range, **p < 0.01

To study the dynamics of sCD18 in the early phase of ethanol-induced liver injury, we employed the NIAAA chronic-binge mouse model. We verified that the mice with this model developed ethanol-induced liver injury in our hands by measuring AST and ALT levels both of which were elevated in ethanol fed mice (Fig. 4a, b). Liver injury was also visible on liver sections (supplementary figure 3). We measured P-sCD18 and observed lower levels in the ethanol-fed mice in comparison with micefed a control diet (Fig. 4c).

## Discussion

Monocytes and macrophages have long been granted a central role in the pathogenesis of alcoholic hepatitis. Yet, our understanding of their involvement is still inadequate and especially human data is scarce. In this study, we report activation of the integrin CD18 on monocytes from patients with alcoholic hepatitis, but diminished shedding per monocyte of the integrins despite elevated P-sCD18. This may be related to direct effects of alcohol or an attenuated anti-inflammatory response to pro-inflammatory mediators.

Previous studies have measured the cellular expression of total CD18, i.e., CD18 integrins in the active and inactive conformations. In patients with Child Pugh C liver cirrhosis and acute liver failure, monocyte expression of CD11b is elevated^4,24,25^. To strengthen the functional understanding of CD18 expression, we now selectively measured ligand-binding active and total CD18 separately^7^. To our knowledge, the strong up-regulation of CD18 activation epitopes, which we here demonstrate, has not previously been reported in human inflammatory disease. In this way, our study adds to a currently limited number of studies relating CD18 integrin conformational change directly to inflammatory disease. This important finding reflects that monocytes in alcoholic hepatitis are primed for binding to adhesion molecules expressed by the endothelium, i.e., ICAM-1.

Soluble CD18 decreases leukocyte adhesion to the endothelium and thereby limits tissue inflammation. The total P-sCD18 pool seems to be a result of shedding from leukocytes and depletion by ligand binding. In chronic rheumatoid arthritis and spondyloarthritis, the plasma levels of sCD18 are decreased and inversely associated with disease activity^13,14^. In very early rheumatoid arthritis and animal models of arthritis induction, the systemic sCD18 levels shows a biphasic temporal pattern with an initial increase in serum sCD18 levels at arthritis induction followed by a secondary decrease. Further, disease remission in early rheumatoid arthritis is associated with increased sCD18 levels^26^. Finally, very high and very low levels of sCD18 levels were recently associated with fatal outcomes in critically ill sepsis patients^27^. Therefore, changes in sCD18 levels seems to be part of the inflammatory response in general. However, the changes seen in AH indicate that sCD18 levels are primarily altered in diseases with involvement of monocytes.

**Fig. 5:**
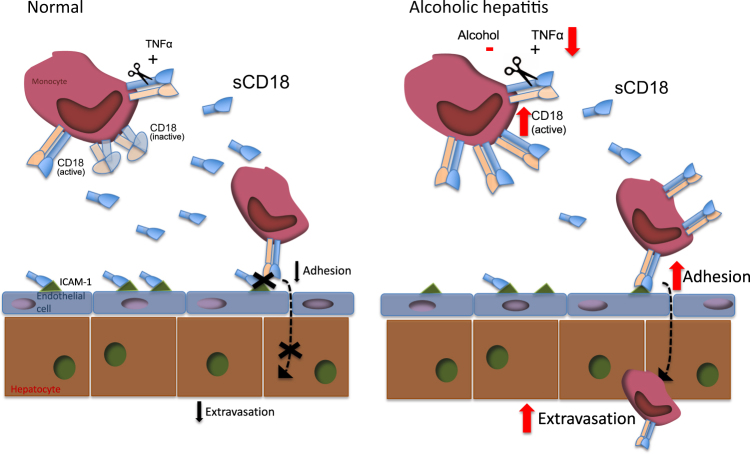
Summary of findings. In the healthy circulation, monocytes express low levels of CD18 receptors in the active conformation, and in response to TNFα, shed CD18, leading to the formation of soluble (s) CD18. The sCD18 complex conceals ICAM-1 on the endothelial surface and thereby limits infiltration of monocytes into the liver. In alcoholic hepatitis, monocytes have a higher expression of active-conformation CD18 receptors. Moreover, ethanol decreases the shedding of the CD18 and fewer CD18 receptors are shed in response to TNFα, whereby the level of sCD18 per single monocyte is decreased in alcoholic hepatitis. This may leave more ICAM-1 receptors available for monocyte adhesion and thus facilitates monocyte extravasation into the liver

Previous studies have shown shedding of CD18 from especially intermediate monocytes and neutrophils^13,15^. In patients with alcoholic hepatitis the shedding of CD18 per leukocyte is decreased despite elevated total P-sCD18. This suggests that the increased monocyte and neutrophil count in alcoholic hepatitis is a likely explanation for the high P-sCD18. Especially, there was an association between high numbers of intermediate monocytes and P-sCD18 levels and in vitro CD18 shedding suggesting that this cell subset is also essential for CD18 shedding in alcoholic hepatitis. The lowered shedding per monocyte, in combination with monocyte CD18 activation, tilts the balance towards adhesion to the endothelium as the first step in extravasation. Consequently, we hypothesize that this may contribute to increased monocyte migration and partly explain the increased number of monocytes in the liver during alcoholic hepatitis (Fig. 5)^13–15,28^. Unfortunately, we do not have liver specimens from these patients to verify that this indeed translates into a large, CD18-positive liver infiltrate. However, the population of intermediate monocytes, which is the predominant sCD18 shedder amongst PBMCs, is also the monocyte population, which increases the most in the liver during inflammation^13,29^. This further supports a dominant role of the CD18 system in recruitment of monocytes to the liver. The decreased total plasma sCD18 level in our mouse model may reflect that shedding of CD18 indeed is low in the early stages of alcoholic liver disease contributing to the initiation of liver inflammation or simply be explained by the fact that it is a model without monocytosis or marked monocyte infiltration in the liver^12^.

The decreased single monocyte shedding of CD18 may arise from direct effects of alcohol, as we observe in vitro the ability of ethanol to lower CD18 shedding. Such dampening effects of ethanol on immune functions have been demonstrated in several studies of other monocyte functions^30,31^. The lowered P-sCD18 observed in our mouse model, where the animals are gavaged with ethanol only 9 h prior to being sacrificed lends support to the ethanol depressing mechanism also being present in vivo. Although most alcoholic hepatitis patients were abstinent from alcohol in the weeks leading up to disease presentation in hospital, monocyte function is only partially restored in subjects with alcohol use disorders after 2 weeks of abstinence. Effects of alcohol consumption are therefore likely still impacting immune functions in our study patients^30^. In response to the pro-inflammatory mediator TNFα, alcoholic hepatitis patients fail to raise sCD18 equivalent to that of healthy subjects. The lack of such anti-inflammatory response is again in accordance  with several studies showing a pro-inflammatory skewing of monocytes in alcoholic liver disease, which aggravates the effects of alcohol on this system^32–34^. This tolerance to TNFα may also explain why we do not observe any differences between the pentoxifyllin-treated and the un-treated patients in the CD18 system, and why excluding either group from our analyses does not change our results. However, we cannot in this design exclude an impact of pentoxifyllin on CD18 shedding as the two groups differ also in disease severities.

The inverse relation between the P-sCD18 and in vitro CD18 shedding per monocyte and disease severity scores are in line with findings from arthritis patients and further supports decreased CD18 shedding being detrimental in alcoholic hepatitis^13,26^. The fact that the uncorrected P-sCD18 levels increase with increasing disease severity most likely reflect that leukocytosis increase with increasing disease severity.

Our study is obviously limited by its sample size. To further elucidate whether our findings reflects alcoholic liver disease per se or is predominantly to be attributed to the inflammation of alcoholic hepatitis, additional control groups are required. Nevertheless, this study provides novel insights into the role of monocyte CD18 integrin expression in alcoholic hepatitis. Twenty years ago, antibodies antagonizing the CD11b/CD18 complex were demonstrated to strongly reduce neutrophil adhesion to inflamed endothelium in vitro^35^. Today, antibodies that blocks T cell integrin receptor binding to mucosal adhesion molecules are used as therapy in inflammatory bowel disease^36,37^. Thus, this study brings forward data on a mechanism that could be utilized as a therapeutic target in alcoholic hepatitis and therefore deserves further investigations into its mechanistic.

In conclusion, we provide data to suggest that the CD18 receptor is involved in recruiting monocytes to the liver in alcoholic hepatitis and that regulation of monocyte adhesiveness such as production of the anti-migration complex sCD18, is dysfunctional. As this relates to disease severity, strategies to influence this system may be an option to reduce morbidity and mortality in alcoholic hepatitis and should be explored.

## Study Highlights

### What is current knowledge

- Alcoholic hepatitis holds high mortality and no targeted treatments exist
- Monocyte liver infiltration is dominant but mechanistically poorly elucidated

### What is new here

- Monocytes are primed for extravasation with marked CD18 integrin activation and decreased shedding
- Alcohol and altered responsiveness to TNFα may be responsible

### Translational impact

- These changes in CD18 may contribute to monocyte liver recruitment in AH
- Consequently the CD18 system may be a therapeutic target to limit liver inflammation

## Electronic supplementary material

Supplementary Figures
