# Correction: Decreased monocyte shedding of the migration inhibitor soluble CD18 in alcoholic hepatitis

**DOI:** 10.1038/s41424-018-0041-4

**Published:** 2018-07-24

**Authors:** Sidsel Støy, Thomas Damgaard Sandahl, Anne Louise Hansen, Bent Deleuran, Thomas Vorup-Jensen, Hendrik Vilstrup, Tue Wenzel Kragstrup

**Affiliations:** 1 Department of Hepatology and Gastroenterology, Aarhus University Hospital, Aarhus, Denmark; 2 Department of Biomedicine, Aarhus University, Aarhus, Denmark; 3 Department of Rheumatology, Aarhus University Hospital, Aarhus, Denmark

Correction to: *Clinical and Translational Gastroenterology*; 10.1038/s41424-018-0022-7; published online 15 June 2018

The PDF and HTML versions of the article have been updated to include the Creative Commons Attribution 4.0 International License information.

