# Supplementary material for: Decreased monocyte shedding of the migration inhibitor soluble CD18 in alcoholic hepatitis
Source: Clin Transl Gastroenterol. 2018 Jun 15;9(6):160. doi: 10.1038/s41424-018-0022-7 (PMC6002386; doi:10.1038/s41424-018-0022-7)
Supplement: Supplementary file 1 — Supplementary Figures [file 41424_2018_22_MOESM1_ESM.docx]

**Decreased monocyte shedding of the migration inhibitor soluble CD18 in alcoholic hepatitis**

Sidsel Støy, Thomas Damgaard Sandahl, Anne Louise Hansen, Bent Deleuran, Thomas Vorup-Jensen, Hendrik Vilstrup, Tue Wenzel Kragstrup.

Supplementary figure 1…………………………………………………………………………… 2

Supplementary figure 2…………………………………………………………………………… 3

Supplementary figure 3…………………………………………………………………………… 4

**Supplementary figure 1. Gating strategy to identify monocyte subsets.**


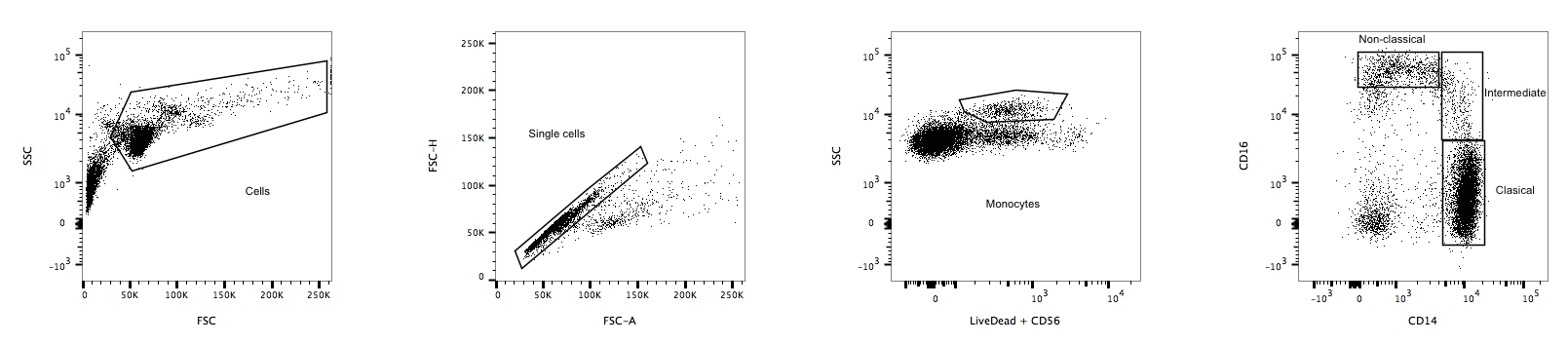


Monocyte subsets were identified amongst peripheral blood mononuclear cells using the above gating strategy. Cells were identified on a forward scatter (FSC) versus side scatter (SSC) plot and doublets were excluded on a FSC-area (A) versus FSC-height (H) plot. A monocyte gate was set on live i.e. Live-Dead negative granular cells that were CD56 negative to avoid contamination from NK cells in the monocyte gate. These monocytes were then subdivided into classical monocytes (CD14^+^CD16^-^), intermediate monocytes (CD14^+^CD16^+^) and non-classical monocytes (CD14^low^CD16^+^).

**Supplementary figure 2. Decreased shedding of CD18 by neutrophils and total leukocytes in alcoholic hepatitis.**

The plasma (P) concentration of sCD18 was measured by TRIFMA in healthy controls and in patients with alcoholic hepatitis at the day of diagnosis and at day 14 and 30 after diagnosis and divided by (A) neutrophil count and (B) total leukocyte count. Alcoholic hepatitis patients are compared with healthy controls, ranksum, bars represent median, interquartile range, *p<0.05, **p<0.01.

**Supplementary figure 3. Liver sections from mouse model.**

Using female C57/BL mice, we employed the chronic-binge ethanol model of alcoholic liver injury. Mice were fed a liquid Lieber-DeCarli diet containing either ethanol or maltose dextrin (control for calories in ethanol) for 10 days and gavaged with ethanol or maltose dextrin at day 10 and sacrificed 9 hours later. Hematoxylin and eosin staining of liver tissue sections from (A) ethanol fed and (B) control fed female C57/BL mice.
